# Association Between Ambient Air Pollution and Cardiac Morpho-Functional Phenotypes: Insights From the UK Biobank Population Imaging Study

**DOI:** 10.1161/CIRCULATIONAHA.118.034856

**Published:** 2018-11-12

**Authors:** Nay Aung, Mihir M. Sanghvi, Filip Zemrak, Aaron M. Lee, Jackie A. Cooper, Jose M. Paiva, Ross J. Thomson, Kenneth Fung, Mohammed Y. Khanji, Elena Lukaschuk, Valentina Carapella, Young Jin Kim, Patricia B. Munroe, Stefan K. Piechnik, Stefan Neubauer, Steffen E. Petersen

**Affiliations:** 1 William Harvey Research Institute, National Institute for Health Research Barts Biomedical Research Centre, Queen Mary University of London, Charterhouse Square, UK (N.A., M.M.S., F.Z., A.M.L., J.A.C., J.M.P., R.J.T., K.F., M.Y.K., P.B.M., S.E.P.).; 2 Barts Heart Centre, St. Bartholomew’s Hospital, Barts Health National Health Service Trust, London, UK (N.A., M.M.S., F.Z., K.F., M.Y.K., S.E.P.).; 3 Division of Cardiovascular Medicine, Radcliffe Department of Medicine, University of Oxford, UK (E.L., V.C., Y.J.K., S.K.P., S.N.).; 4 Clinical Pharmacology, William Harvey Research Institute, Barts and The London School of Medicine and Dentistry, Queen Mary University of London, UK (P.B.M.).; 5 Department of Radiology, Severance Hospital, Yonsei University College of Medicine, Seoul, South Korea (Y.J.K.).

**Keywords:** air pollution, magnetic resonance imaging, phenotypes

## Abstract

**Background:**

Exposure to ambient air pollution is strongly associated with increased cardiovascular morbidity and mortality. Little is known about the influence of air pollutants on cardiac structure and function. We aim to investigate the relationship between chronic past exposure to traffic-related pollutants and the cardiac chamber volume, ejection fraction, and left ventricular remodeling patterns after accounting for potential confounders.

**Methods:**

Exposure to ambient air pollutants including particulate matter and nitrogen dioxide was estimated from the Land Use Regression models for the years between 2005 and 2010. Cardiac parameters were measured from cardiovascular magnetic resonance imaging studies of 3920 individuals free from pre-existing cardiovascular disease in the UK Biobank population study. The median (interquartile range) duration between the year of exposure estimate and the imaging visit was 5.2 (0.6) years. We fitted multivariable linear regression models to investigate the relationship between cardiac parameters and traffic-related pollutants after adjusting for various confounders.

**Results:**

The studied cohort was 62±7 years old, and 46% were men. In fully adjusted models, particulate matter with an aerodynamic diameter <2.5 µm concentration was significantly associated with larger left ventricular end-diastolic volume and end-systolic volume (effect size = 0.82%, 95% CI, 0.09–1.55%, *P*=0.027; and effect size = 1.28%, 95% CI, 0.15–2.43%, *P*=0.027, respectively, per interquartile range increment in particulate matter with an aerodynamic diameter <2.5 µm) and right ventricular end-diastolic volume (effect size = 0.85%, 95% CI, 0.12–1.58%, *P*=0.023, per interquartile range increment in particulate matter with an aerodynamic diameter <2.5 µm). Likewise, higher nitrogen dioxide concentration was associated with larger biventricular volume. Distance from the major roads was the only metric associated with lower left ventricular mass (effect size = −0.74%, 95% CI, −1.3% to −0.18%, *P*=0.01, per interquartile range increment). Neither left and right atrial phenotypes nor left ventricular geometric remodeling patterns were influenced by the ambient pollutants.

**Conclusions:**

In a large asymptomatic population with no prevalent cardiovascular disease, higher past exposure to particulate matter with an aerodynamic diameter <2.5 µm and nitrogen dioxide was associated with cardiac ventricular dilatation, a marker of adverse remodeling that often precedes heart failure development.

## Abstract 2

Supplemental Digital Content is available in the text.

**Clinical Perspective**

**What Is New?**

- Although ambient air pollutants are known to be associated with increased cardiovascular morbidity and mortality, limited information is available on the link between air pollutants and cardiac structure and function.
- In this cross-sectional analysis of a large population free from pre-existing cardiovascular disease, higher past exposure to fine particulates with an aerodynamic diameter <2.5 µm and nitrogen dioxide were associated with larger cardiac biventricular volumes, which is a well-recognized pathophysiological adaptation, heralding heart failure development.
- Proximity to major roads, a surrogate for chronic air pollution exposure, was additionally associated with higher left ventricular mass, which is known to portend adverse outcomes.

**What Are the Clinical Implications?**

- The association between ambient air pollution and adverse cardiac phenotypic changes in individuals without prevalent cardiovascular disease suggests that air pollution should be recognized as a major modifiable risk factor that needs to be targeted via public health measures.
- These cardiac morphological alterations are apparent despite relatively low exposure levels meeting the current air quality standards, making a strong case to double efforts to control emission of the noxious pollutants.

The deleterious effect of air pollutants on cardiovascular health is well established. Several studies have demonstrated strong associations between exposure to air pollution and increased risks of coronary artery disease, heart failure, stroke, cardiovascular mortality, and all-cause mortality.^1^ Traffic-related environmental pollution consists of a complex mixture of gaseous and particulate components, alongside auxiliary elements such as noise and psychological stress. Among all air pollutants, particulate matter (PM) pollution—specifically fine particulates with an aerodynamic diameter <2.5 µm (PM_2.5_)—has repeatedly been associated with cardiovascular morbidity and mortality. Inhalation of PM_2.5_ can initiate and sustain physiological and biochemical changes through elevation of pulmonary and systemic inflammatory and oxidative stress, autonomic imbalance, endothelial dysfunction, hypertension, atherosclerosis, and thrombosis, which are all key substrates for adverse cardiac remodeling leading to detrimental outcomes.^2^

Cardiac morpho-functional parameters are prognostically important biomarkers in health and disease. Left ventricular (LV) mass, for example, is a well-recognized predictor of cardiovascular morbidity and mortality even in individuals without established cardiovascular disease (CVD).^3^ LV geometric patterns and the morpho-functional indices of other cardiac chambers also carry prognostic information in the setting of CVD.^4–11^ Although the associations between ambient air pollutants and increased incidence of myocardial infarction and heart failure have been established,^12,13^ there is a paucity of information in the current literature about the influence of air pollution on cardiac structure and function. Determining the impact of individual air pollutants on cardiac phenotypes is challenging for several reasons owing to socioeconomic confounders, relatively small effect sizes, and the variability of exposure and outcome measurement techniques.

The UK Biobank is a large-scale prospective cohort study of half a million people aged 40 to 69 years. In addition to a rich repository of information on demographics, risk factors, and environmental exposure data, a subgroup of UK Biobank participants undergo deep phenotyping with cardiovascular magnetic resonance (CMR), which is the reference imaging modality for quantification of the cardiac structural phenotypes.^14^ In this study, we aim to explore the association between chronic past exposure to traffic-related ambient air pollution and the cardiac imaging parameters after accounting for various potential confounders in the UK Biobank cohort. We hypothesized that annual average air pollutants and other traffic-related factors quantified approximately 5 years before cardiac imaging have a detectable adverse association with cardiac imaging phenotypes in individuals free from known cardiovascular disease.

## Methods

### Data Access

The data, analytic methods, and study materials will be returned to the UK Biobank. The UK Biobank will make these data available to all bona fide researchers for all types of health-related research that is in the public interest, without preferential or exclusive access for any person. All researchers will be subject to the same application process and approval criteria as specified by the UK Biobank. Please see the UK Biobank’s website for the detailed access procedure (http://www.ukbiobank.ac.uk/register-apply/).

### Study Population

The UK Biobank is a large population-based, prospective cohort study which has collected a wealth of information on health and lifestyle data, physical measurements, biological samples, and cardiac phenotypes derived from CMR. This ambitious project aims to provide resources to disentangle the genetic and environmental determinants of complex diseases affecting middle and old age. The study protocol has been described in detail previously.^15^ In brief, ≈9.2 million UK residents aged between 40 to 69 years, who were registered with the UK National Health Service and living <25 miles from 1 of the 22 study assessment centers, were invited to join the study. Among those who responded to the invitation, >500 000 people were enrolled from 2006 through 2010. The sample size of 500 000 was calculated a priori for reliable detection of the effects of different exposures on a wide variety of conditions in nested case-control studies. Although the UK Biobank cohort is not designed to be representative of the UK general population (because of healthy volunteer selection bias), it is well suited to study exposure-disease relationships because of its large size and heterogeneity of exposure measures.^16^ The baseline summary characteristics of the cohort can be viewed in the data showcase on UK Biobank’s website (www.ukbiobank.ac.uk). The CMR imaging substudy was commenced in 2014, and this study included the first 5065 consecutive participants who returned for imaging enhancement in 2014 to 2015. The study complies with the Declaration of Helsinki and was approved by our institutional review body. All participants provided informed written consent. The UK Biobank’s scientific protocol and operational procedures were approved by the Northwest Research Ethics Committee in the UK.

### Ambient Air Pollution, Noise, and Traffic Exposure

The annual average concentration of PM_2.5_, PM with an aerodynamic diameter of less than 10 µm (PM_10_), PM with an aerodynamic diameter between 2.5 and 10 µm (PM_coarse_), PM_2.5_ absorbance (a measurement of the blackness of PM_2.5_ filter – a proxy for elemental or black carbon), nitrogen dioxide (NO_2_), and nitrogen oxides (NO_x_) were calculated centrally by the UK Biobank using a Land Use Regression model developed by the ESCAPE project.^17,18^ Land Use Regression models calculate the spatial variation of annual average air pollutant concentration at participants’ home addresses given at the baseline visit, using the predictor variables obtained from the Geographic Information System such as traffic, land use, and topography. Because NO_2_ and PM_10_ annual concentration data were available for several years (2005, 2006, 2007, and 2010 for NO_2_ and 2007 and 2010 for PM_10_), we averaged the values to get the mean estimate. All other particulate matter and nitrogen pollutants had the exposure data for a single year (2010). The median leave-one-out cross-validated variance explained by the model was 71% for PM_2.5_, 77% for PM_10_, 68% for PM_coarse_, 89% for PM_2.5_ absorbance, 82% for NO_2_, and 78% for NO_x_.

Average exposure to noise for year 2009 was estimated from a model based on common noise assessment methods in Europe (CNOSSOS-EU).^19^ This technique allows large-scale noise mapping for epidemiological studies using data on traffic flow, speed and composition, land cover, building heights, road network, air temperature, and wind direction. Noise pollution was represented by 24-hour (daily) sound pressure level (A-weighted sound level in decibels) averaged over 1 year as suggested by the World Health Organization.^20^

Traffic intensity on the nearest major road was defined as the total number of motor vehicles per 24 hours, averaged over the course of 1 year. The traffic count and road network data were provided by the UK Department for Transport and the Ordnance Survey Meridian 2 (OSM2) road network (scale 1:50 000, 1 meter accuracy) in year 2009. Proximity to traffic was characterized by the distance from home address to the nearest major road.

### CMR Parameters

The detailed CMR protocol and analysis methods have been described previously.^21^ In brief, all CMR studies were acquired with a wide-bore 1.5 Tesla scanner (MAGNETOM Aera, Syngo Platform VD13A, Siemens Healthcare, Erlangen, Germany), and analyses were performed using cvi42 postprocessing software (Version 5.1.1, Circle Cardiovascular Imaging Inc, Calgary, Canada). LV mass and volumes, right ventricular (RV) volumes, and left atrial (LA) and right atrial volumes were manually measured from balanced steady-state free precession cine short- and long-axis images. The following cardiac phenotypes were included: LV end-diastolic volume (EDV), LV end-systolic volume (LV ESV), LV ejection fraction (EF), LV mass, RV EDV, RV ESV, RV EF, LA maximal volume, LA EF, right atrial maximal volume, right atrial EF, and LV geometric remodeling patterns. The LV geometric remodeling patterns were classified according to LV mass indexed to body surface area and LV mass to end-diastolic volume ratio (LVMVR, CMR-equivalent of relative wall thickness) as previously described.^22^ Normal cut-off values for LV mass and LVMVR were obtained from the 95% prediction intervals of sex-specific reference ranges.^21^ Four distinct LV geometric remodeling patterns were defined: (1) normal (normal indexed LV mass and LVMVR), (2) concentric remodeling (normal indexed LV mass and increased LVMVR), (3) eccentric hypertrophy (increased indexed LV mass and normal LVMVR), and (4) concentric hypertrophy (increased indexed LV mass and increased LVMVR).

### Statistical Analyses

Because air pollution estimates were modeled using participants’ home address given at the baseline visit, we restricted the data analysis to those who remained at the same address between the baseline and imaging visits. We also excluded individuals with any known cardiovascular disease based on the self-reported questionnaires and hospital episode data to mitigate the potential impact of established cardiac conditions on the imaging parameters. All continuous variables were assessed for normality using histograms and quantile-quantile plots. Natural logarithmic transformation was performed on non-Gaussian dependent variables where possible. Descriptive statistics for continuous variables are presented as mean (SD) or median (interquartile range [IQR]), whereas categorical variables are presented as number (percentage).

We imputed missing data by multiple imputation by chained equations approach to create 50 complete datasets.^23^ We used predictive mean matching for continuous variables, logistic regression for binary variables, and polytomous regression for categorical variables. All covariates and interaction terms were included in the imputation models. The maximum iteration was set at 50, and convergence was confirmed by visual examination of trace plots.

We constructed separate multiple linear regression models to examine the associations between each air pollutant and continuous cardiac CMR variables. For categorical LV geometric remodeling patterns, we used multinomial logistic regression to model the effect of pollution. In all statistical models, we adjusted for the following: (1) demographics—age at imaging visit, sex, and ethnicity; (2) anthropometrics—height and body mass index; (3) socioeconomic factors—average household income, employment status, Townsend deprivation index, and educational attainment; (4) cardiac risk factors—systolic blood pressure, diastolic blood pressure, heart rate, smoking status, regular alcohol use, hypertension, diabetes mellitus, and respiratory disease; (5) medications—antihypertensive medication, lipid-lowering medication, and insulin; and (6) physical activity—7-day average acceleration from accelerometer. All covariates were chosen a priori for their established or presumed influence on the cardiovascular structure and function. The measurement protocols and covariate definitions are provided in the Definitions of Covariates section in the online-only Data Supplement. The β-coefficients (effect estimates) of log-transformed variables were antilogged and expressed as percentage change. The mean estimates and standard errors of the β-coefficients for the imputed datasets were combined with Rubin’s rules (see Methods in the online-only Data Supplement).^24^ Because we have scaled all pollutants by their respective IQR before entering into the regression models, their effect estimates represent the change in dependent CMR variable per IQR increment in pollutant.

We conducted the following secondary analyses: (1) an analysis of effect modification by age, sex, and smoking status by introducing cross-product terms; (2) an analysis excluding hypertension, diabetes, systolic blood pressure, diastolic blood pressure, and heart rate because of their potential mediating effects on the relationship between air pollution and cardiac phenotypes; (3) an analysis of the confounding effects of noise and proximity to traffic on the significant associations between air pollutants and cardiac measurements; and (4) an analysis of cases with clinically unrecognized myocardial infarction (MI) based on the evaluation of CMR images. Cases with possible MI were first selected by identifying thin left ventricular myocardial segments (end-diastolic wall thickness <5.5 mm for the basal and midsegments) and possible regional hypokinesis (systolic wall thickening—end-systolic wall thickness minus end-diastolic wall thickness—of <2 mm) as recommended by Baer et al.^25,26^ These cases were then manually evaluated by 3 analysts with significant experience in reporting clinical CMR studies (M.Y.K., N.A., both European Association of Cardiovascular Imaging CMR level 3-certified cardiologists, and K.F., with 4 years’ experience in reporting clinical CMR studies).

Sensitivity analyses were conducted by (1) restricting the sample to participants with complete data, (2) indexing continuous CMR-derived phenotypes by height^2.7^, and (3) restricted cubic spline transformation of exposure variables to investigate nonlinear relationships. The optimal number of knots for restricted cubic spline–transformed variables was determined by the Akaike information criterion. Nonlinearity was assessed with the ANOVA *F* statistics and visualized with line plots. The regression model assumptions were checked with residuals plots. A *P* value of <0.05 was considered significant. Multiple imputation, multinomial regression, and restricted cubic spline transformation were performed using the mice, nnet, and rms packages, respectively.^27–29^ We used R (version 3.4.3) for all statistical analyses.^30^

## Results

### Baseline Demographics

**Figure 1.**
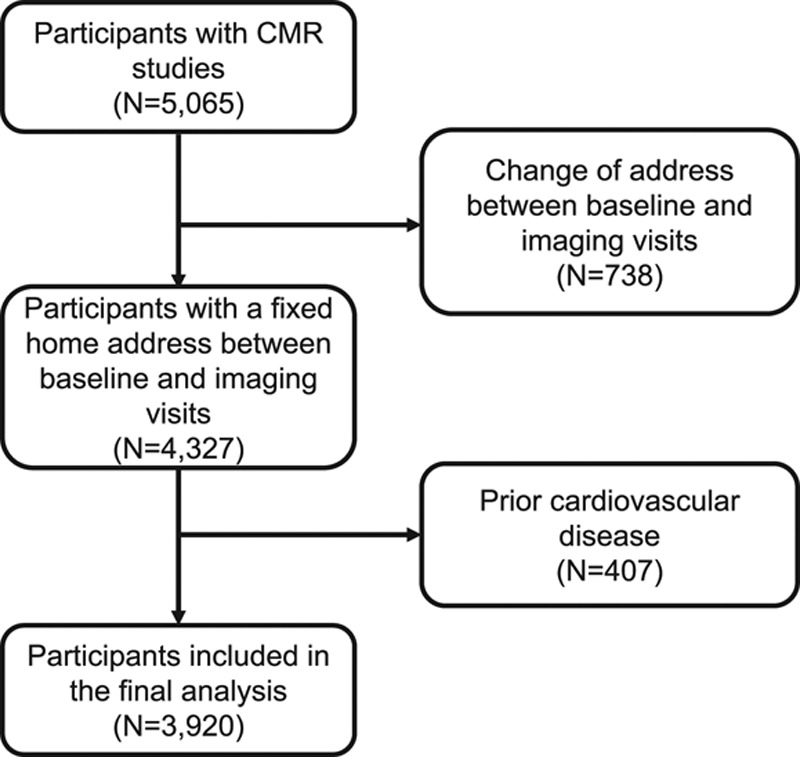
**Case selection flowchart.** CMR indicates cardiovascular magnetic resonance.

A total of 5065 individuals were considered for this study. Of these, we excluded 738 individuals who had moved home between the baseline and imaging visit. A further 407 individuals were excluded because of pre-existing CVD—highest prevalent CVD was coronary artery disease (n [%] = 198 [4.6%]), resulting in 3920 individuals included in the final analysis (Figure 1). The baseline characteristics of the final cohort are presented in Table 1. The mean age of the cohort was 61.7 years, and 45.6% were men. The median (IQR) annual average concentration of the two main pollutants, PM_2.5_ and NO_2_, were 9.9 (1.32) µg/m^3^ and 28.2 (11.4) µg/m^3^, respectively. The median (IQR) duration between the year of exposure estimate and the imaging visit was 5.2 (0.6) years. There was no clinically significant difference in characteristics between the whole cohort and complete cases without missing data (Table I in the online-only Data Supplement).

**Table 1.**
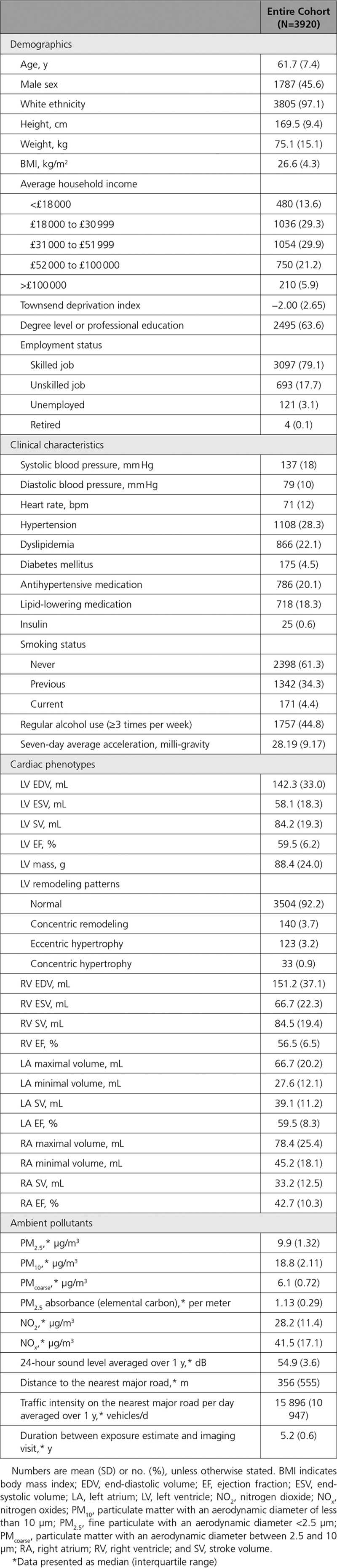
Participant Characteristics

### Relationship Between Particulate Matter Pollutants and Cardiac Phenotypes

**Figure 2.**
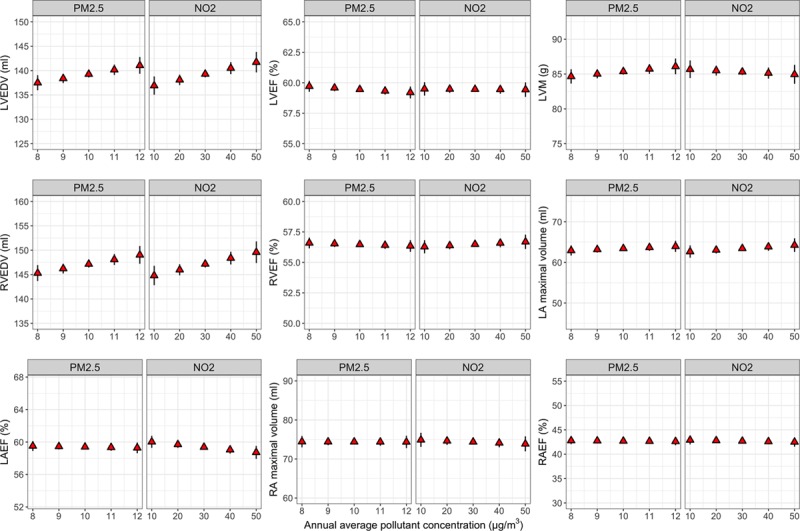
**Association between annual average concentrations of fine particulate matter (PM**_**2.5**_**) and nitrogen dioxide (NO**_**2**_**) and cardiac parameters.** The figure shows the marginal means (with 95% CI) of cardiac parameters at different levels of PM_2.5_ and NO_2_ concentrations. Marginal means were estimated from the linear regression models adjusted for all covariates. Intervals of pollutant concentrations (*x* axis) were chosen to closely represent the range of pollutant concentration observed in the cohort. Higher levels of PM_2.5_ and NO_2_ were associated with larger LV EDV and RV EDV. No significant association was observed between air pollutants and other cardiac parameters. EDV indicates end-diastolic volume; EF, ejection fraction; LA, left atrium; LV, left ventricle; LVM, left ventricular mass; RA, right atrium; and RV, right ventricle.

The associations between particulate matter pollutants and cardiac phenotypes are presented in Table 2 and Figure 2. After adjustment for all covariates, PM_2.5_ concentration was significantly associated with larger biventricular volume (effect size for LV EDV = 0.82%, 95% CI, 0.09–1.55%, *P*=0.027; effect size for LV ESV = 1.28%, 95% CI, 0.15–2.43%, *P*=0.027; effect size for RV EDV = 0.85%, 95% CI, 0.12–1.58%, *P*=0.023, per IQR increment in PM_2.5_ concentration). Likewise, PM_10_ had identical association patterns with slightly smaller magnitude of effect sizes per IQR increment. Neither PM_2.5_ nor PM_10_ was associated with other cardiac parameters and LV geometric remodeling patterns. PM_coarse_ and PM_2.5_ absorbance did not have any association with the cardiac phenotypes.

**Table 2.**
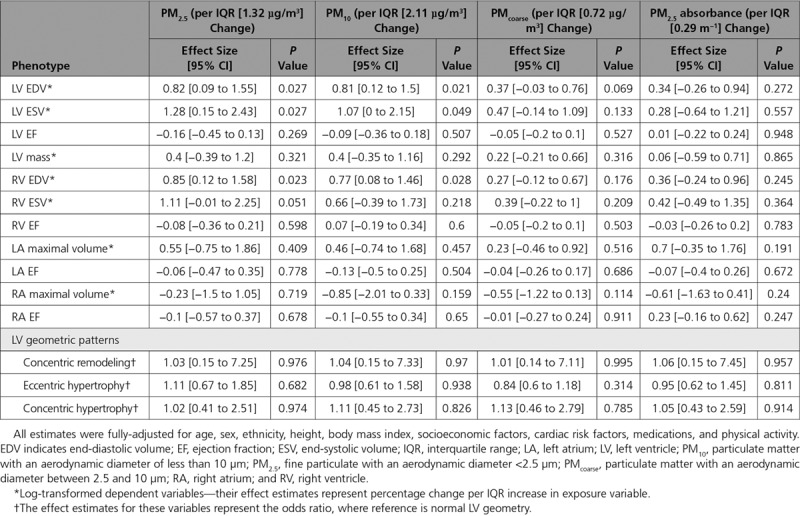
Associations Between Annual Average Particulate Matter Concentration and Cardiac Phenotypes

### Relationship Between Oxides of Nitrogen and Cardiac Phenotypes

Table 3 presents the relationships between nitrogen pollutants and cardiac parameters after adjustment for all covariates. Higher NO_2_ concentration was significantly correlated with larger LV EDV and RV EDV (effect size for LV EDV = 0.91%, 95% CI, 0.12–1.7%, *P*=0.025; effective size for RV EDV = 0.85%, 95% CI, 0.06–1.65%, *P*=0.035, per IQR increment in NO_2_ concentration; Figure 2). However, NO_x_ had no significant association with CMR-derived measurements.

**Table 3.**
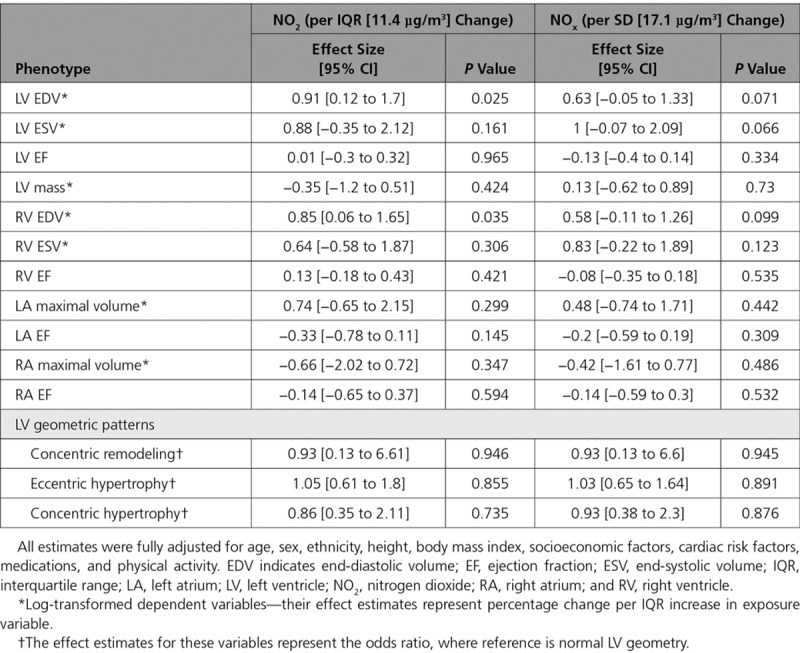
Associations Between Annual Average Nitrogen Dioxide and Nitrogen Oxides Concentration and Cardiac Phenotypes

### Relationship Between Noise, Road Traffic Factors, and Cardiac Phenotypes

The associations between noise, distance to the nearest major road, and traffic intensity and CMR-derived phenotypes are detailed in Table 4. Being exposed to higher ambient sound level was associated with larger LV ESV (effect size = 0.69%, 95% CI, 0.03–1.35%, *P*=0.041, per IQR increment in 24-hour sound level averaged over 1 year). Interestingly, in addition to the significant association with biventricular volume, living further away from major roads was also associated with lower LV mass (effect size = −0.74%, 95% CI, −1.3 to -0.18%, *P*=0.01, per IQR increment in distance to major roads). There was no significant relationship between traffic intensity and any of the cardiac morpho-functional phenotypes.

**Table 4.**
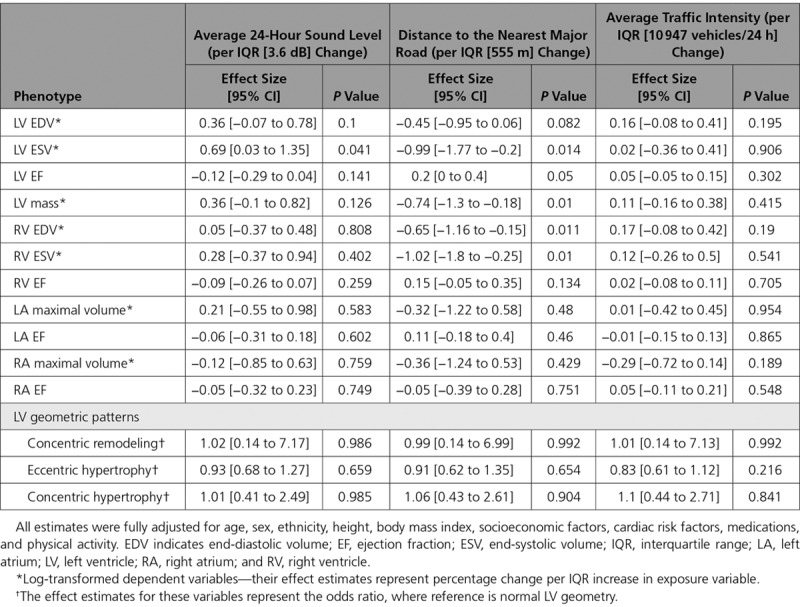
Associations Between Annual Average 24-Hour Sound Level, Distance to Nearest Major Road, and Annual Average Traffic Intensity on the Nearest Major Road Over 24-Hour and Cardiac Phenotypes

### Effect Modification and Mediator Analyses

We investigated whether age, sex, and smoking status modify the significant relationships between each pollutant and cardiac phenotypes. Smoking status was the only important effect modifier, where being a current smoker significantly enhanced the positive association between PM_10_ concentration and RV EDV (effect size difference = +3.3% for current smoker compared to nonsmoker, 95% CI, 0.06–6.7%, *P*=0.046). The regression models that were not adjusted for hypertension, diabetes mellitus, systolic blood pressure, diastolic blood pressure, and heart rate produced results with similar magnitude in effect size when compared with the fully adjusted models (Tables II through IV in the online-only Data Supplement).

The wall motion assessment to determine unrecognized MI cases revealed 600 cases with possible regional wall motion abnormalities based on cut-off values of LV end-diastolic wall thickness <5.5 mm and LV systolic wall thickening of <2 mm. After manual evaluation, we discovered 43 CMR studies with truly hypokinetic LV segments (10 studies with global hypokinesis and 33 studies with regional hypokinesis). The distribution of hypokinetic segments in the 33 cases with regional wall motion abnormalities is displayed in Figure I in the online-only Data Supplement. We observed that mid to apical inferior and lateral segments were most commonly affected. Because of the limited number of cases with probable unrecognized MI based on cine CMR data (<1% of the entire cohort), we were not able to assess the relationship between air pollution and unrecognized or unreported MI.

We found that the associations between PM_2.5_ and NO_2_ concentrations and LV volume were not independent of noise or distance to major roads and vice versa (ie, the relationships between noise/distance to major roads and LV volume were also confounded by PM_2.5_ and NO_2_). The associations between PM_2.5_ and NO_2_ and RV volume were independent of noise but not from distance to major roads.

### Sensitivity Analyses

Sensitivity analyses including only participants with complete data gave no materially different results. Equivalently, fitting the models with continuous CMR parameters indexed to height^2.7^ produced similar findings. There was no consistent evidence to support nonlinear relationships between air pollutants and cardiac measurements (Figure II in the online-only Data Supplement). The fitted model diagnostic plots showed no evidence of heteroscedasticity or deviation from normality of residuals.

## Discussion

In a cross-sectional investigation of 3920 individuals free from known cardiovascular disease, this study identified the following important findings: (1) higher concentrations of PM_2.5_ and NO_2_ were associated with biventricular enlargement; (2) the lack of association between PM_coarse_ and cardiac phenotypes suggests that the association between PM and cardiac chamber size was predominantly driven by the finer particles; (3) other environmental stressors such as noise pollution and proximity to major roads were also correlated with LV dilatation; (4) among all traffic-related factors, only proximity to major roads was predictive of higher LV mass; and (5) no perceptible difference in traditional LV geometric remodeling pattern in relation to differing air pollutant concentration was found.

Accumulating evidence based on meta-analyses indicates an increased risk of heart failure hospitalization associated with higher PM_2.5_ and NO_2_ exposure (1.28% increase in risk per 10 μg/m^3^ increase in PM_2.5_ and 1.7% increase in risk per 10 parts per billion increase in NO_2_).^13,31^ However, the connection between air pollution and cardiac remodeling, which is likely to precede the development of heart failure by months, had not received an in-depth investigation. Previous studies in this arena have typically examined a limited number of pollutants or cardiac phenotypes,^32–34^ animal models,^35,36^ or had relatively small sample sizes.^37,38^ Our study is the largest single epidemiological study to date that investigated the association between chronic exposure to several traffic-related pollutants and cardiac structural variations using highly precise and reproducible CMR measurements, which further enhanced the statistical power.

This is the first study to report the association between PM_2.5_ and NO_2_ concentration and LV dilatation—an ominous sign that often heralds cardiac decompensation—in a population free from pre-exiting cardiovascular disease.

### Association Between Ambient Pollutants and Cardiac Parameter—Summary of Evidence

The findings from this study should be interpreted in the context of the currently available evidence in animal and human studies. In a controlled-exposure study with mice, prolonged exposure to concentrated PM_2.5_ (mean exposure chamber concentration of 85.3 µg/m^3^) led to increased LV dimensions, decreased fractional shortening, and reduction in contractile reserve to dobutamine.^36^ Similarly, in utero and early life exposure to concentrated PM_2.5_ in mice appeared to increase LV cavity size and impair LV function with histological evidence of cardiac collagen deposition.^35,39^ A human study that assessed the cross-sectional association between residential air pollution and cardiac measurements derived from echocardiogram in 671 white Europeans found a reduction in LV longitudinal strain and strain rate with higher levels of PM_2.5_, PM_10_, NO_2_, and black carbon.^38^ Similar to our study, no association was found between the ambient pollutants and the LA volume, LV mass, or LV EF. However, in contrast to our study, they found no correlation between LV dimension and the ambient pollutants, which could be explained by the well-recognized limitation of 2-dimensional echocardiogram in measurement of LV dimensions and the study being significantly underpowered. The SALIA cohort with 264 elderly women (mean age of 74.4 years) reported some signals of association between larger indexed LA volume and higher PM_2.5_, NO_2_, and NO_x_ exposure.^37^ The lack of association between LA size and pollution concentration in our study could be attributable to a much lower level of exposure (mean PM_2.5_ of 9.86 µg/m^3^ in our cohort versus 17.4 µg/m^3^ in the SALIA cohort) and younger age of the participants.

Another echocardiogram-based study by Weaver et al^34^ in 4866 African-American individuals (JHS) did not find any association between the distance to major roads and LV EF and LA diameter index, in parallel to our results. Intriguingly, they reported a small increase in pulmonary artery systolic pressure in those living 300 to 999 meters from major roads (compared with those who lived ≥1000 meters)—a finding which may explain the mechanism of RV dilatation in relation to the distance to major roads observ ed in our study. A follow-up study in the same JHS cohort found a 1.2-mm larger LV end-systolic diameter in participants residing <150 meters from a major road in comparison with those living ≥1000 meters), although no association was detected between indexed LV mass and proximity to major roads.^40^

Perhaps the most comparable study to-date was conducted in the MESA cohort (sample size of 3827; age 45–84 years), which also underwent CMR imaging.^32^ Interestingly, they only investigated the impact of PM_2.5_ and proximity to traffic on LV EF and LV mass. In their fully adjusted models, living within 50 m of a major road was associated with higher indexed LV mass, whereas PM_2.5_ did not influence LV mass or LV EF; these results are consistent with our findings. Another MESA study by Leary and colleagues^33^ that explored the relationship between NO_2_ and NO_x_ exposure and RV phenotypes observed a small increase in RV mass and RV EDV per IQR increase in NO_2_; the latter finding was replicated in our study, but RV mass was not available in our cohort for comparison.

All available epidemiological evidence to date, including our findings, suggests that ambient particulate and nitrogen pollutants predominantly affect the ventricular chamber size and possibly the long-axis function, while exerting a minimal influence on other cardiac indices such as LV radial function or LA size. Residential proximity to major roadways is regarded as a surrogate for long-term exposure to traffic-related pollutants and has been known to be associated with adverse cardiovascular and pulmonary outcomes.^41^ Unlike the ambient pollutants, it is associated with higher LV mass, a well-recognized cardiovascular prognosticator, which could be attributable to the contributions from unmeasured noxious elements (such as sympathetic stimulation from stress and annoyance) and residual confounding from latent socioeconomic factors. Given the known links between coronary artery disease and air pollution, the effect estimates of the associations between air pollutants and cardiac parameters in our study are likely to be conservative because of a priori exclusion of individuals with pre-existing CVD.

### Biological Mechanisms Mediating Cardiac Remodeling

Air pollution exposure is known to be associated with elevation of oxidative stress, immune-mediated systemic inflammation, and hypercoagulation, which can induce atherosclerosis, myocardial ischemic damage, and associated cardiac remodeling.^42–45^ Indeed, ventricular enlargement in association with PM_2.5_ and NO_2_ in our cohort free from known cardiovascular disease could be attributable to adverse remodeling secondary to unrecognized or silent MI. In our study, the prevalence of probable MI based on cine CMR data was low (<1% of the entire cohort). Although the true prevalence of silent MI is likely to be higher, we were unable to ascertain subtle subendocardial infarction in the absence of late gadolinium contrast enhancement images. The population prevalence of unrecognized MI was previously reported to be 16.7% in an Icelandic cohort,^46^ although the latter imaged a much older population (mean age of 76.7 years versus 61.7 years in our study) and had benefited from augmented sensitivity and specificity afforded by the aforementioned contrast agent. Another potential contributing mechanism is through vasoconstriction and systemic hypertension attributable to a combination of endothelial dysfunction and autonomic imbalance. However, in our study, systolic and diastolic components of blood pressure and presence of hypertension do not appear to mediate the association between air pollution and cardiac parameters, suggesting that oxidative stress is likely to be predominantly responsible for cardiac phenotypic alterations which often precede clinical heart failure.

### Strength and Limitations

Our study is the first to report the deleterious influence of a wide range of ambient pollutants on prognostically important cardiac chamber size in humans free from any pre-existing cardiovascular disease. The strengths of this study include a large sample size, highly accurate and reproducible measurements by CMR imaging, and uniform data collection protocols, which increase the precision of effect estimates. Our study has a number of limitations. First, we used estimated outdoor pollution at participants’ home address, which does not take into account (1) individual activity pattern such as time spent at home or in traffic, (2) degree of pollutant infiltration into buildings, and (3) indoor air pollution and workplace exposure. Second, biomarkers of oxidative damage, such as malondialdehyde, 4-hydroxy-2-nonenal, 4-oxo-2-nonenal, and acrolein, were not measured in our cohort, which prevented us from validating our findings mechanistically. Third, multiple testing correction was not performed for the regression models. However, the inflation of type I error may be somewhat diminished, although not completely removed, by correlation within exposure and outcome variables: ≈70% of ambient air pollutants (exposure) and directly measured CMR variables (outcome) were at least moderately intercorrelated (Pearson correlation coefficient [*r*]>0.5), and ≈30% of both exposure and outcome variables were significantly correlated (Pearson r>0.7). Finally, the intrinsic weaknesses of the cross-sectional study design mean that the findings should be interpreted with caution while corroborating longitudinal data are pending.

### Clinical Implications

The current European standard of acceptable annual PM_2.5_ concentration is <25 µg/m^3^, whereas the World Health Organization air quality guidelines stipulate a more stringent long-term target of 10 µg/m^3^.^47,48^ Although the relatively low average concentration level in our study population not only achieves the World Health Organization target but surpasses the current European standard by a significant margin, we observed a detectable cardiac remodeling effect which usually heralds detrimental outcomes. Although the effect sizes found in our analyses are relatively small, they are comparable with the impact of other cardiovascular risk factors on cardiac phenotypes (for example, a previous study in the same cohort reported a 2% larger LV ESV per SD [18.1 mm Hg] increase in systolic blood pressure versus a 1.28% larger LV ESV per IQR [1.32 µg/m^3^] increase in PM_2.5_ concentration in this study).^49^

## Conclusions

In this large UK-wide middle-aged population, we found a significant association between higher annual average PM_2.5_ and NO_2_ concentration and larger biventricular volume, which is a hallmark of adverse cardiac remodeling. These cardiac structural alterations in the absence of known cardiovascular disease alludes to a silent pathophysiological adaptation that should be monitored and targeted for treatment. Our findings add to the growing evidence of the damaging effects of ambient pollution even in the setting of relatively low exposure levels. Efforts to reduce air pollutant emission should be prioritized accordingly in public health initiatives and legislative measures.

## Acknowledgments

This research has been conducted using the UK Biobank Resource under Application 2964. We thank all UK Biobank participants and staff.

## Sources of Funding

Drs Peterson, Neubauer, and Piechnik acknowledge the British Heart Foundation for funding the manual analysis to create a cardiovascular magnetic resonance imaging reference standard for the UK Biobank imaging resource in 5000 CMR scans (PG/14/89/31194). Dr Aung is supported by a Wellcome Trust Research Training Fellowship (203553/Z/16/Z). Drs Lee and Petersen acknowledge support from the National Institute for Health Research Barts Biomedical Research Center and from the “SmartHeart” Engineering and Physical Sciences Research Council program grant (EP/P001009/1). Drs Neubauer and Petersen are supported by the Oxford National Institute for Health Research Biomedical Research Center and the Oxford British Heart Foundation Center of Research Excellence. This project was enabled through access to the Medical Research Council eMedLab Medical Bioinformatics infrastructure, supported by the Medical Research Council (grant No. MR/L016311/1). Dr Fung is supported by The Medical College of Saint Bartholomew’s Hospital Trust, an independent registered charity that promotes and advances medical and dental education and research at Barts and The London School of Medicine and Dentistry. The UK Biobank was established by the Wellcome Trust medical charity, Medical Research Council, Department of Health, Scottish Government, and the Northwest Regional Development Agency. It has also received funding from the Welsh Assembly Government and the British Heart Foundation.

## Disclosures

Dr Petersen provides consultancy to Circle Cardiovascular Imaging Inc, Calgary, Canada. The other authors report no conflicts.

## Supplementary Material
