# Supplementary material for: Association Between Ambient Air Pollution and Cardiac Morpho-Functional Phenotypes: Insights From the UK Biobank Population Imaging Study
Source: Circulation. 2018 Nov 12;138(20):2175–86. doi: 10.1161/CIRCULATIONAHA.118.034856 (PMC6250297; doi:10.1161/CIRCULATIONAHA.118.034856)
Supplement: Supplementary file 1 [file cir-138-2175-s001.pdf]

SUPPLEMENTAL MATERIAL

Supplemental Methods

Definitions of covariates

Height was measured with a Seca 202 device (Seca, Birmingham, UK). Body mass index was calculated by dividing weight (kg) with height^2 (m^2). Systolic blood pressure, diastolic blood pressure and heart rate were defined as the average of two consecutive measurements taken with an Omron 705 IT electronic blood pressure monitor (OMRON Healthcare Europe, Hoofddorp, The Netherlands). Educational attainment variable was dichotomised into degree level or professional qualification vs. other qualifications. Regular alcohol use was defined as consumption of alcohol at least three times per week.

Diabetes status and history of respiratory and cardiovascular diseases were ascertained from a combination of participants' response to the health questionnaire and verbal interview at the UK Biobank assessment centre. Respiratory diseases were defined as asthma, chronic obstructive airways disease (COPD), emphysema, chronic bronchitis, bronchiectasis, interstitial lung disease, asbestosis, pulmonary fibrosis, fibrosing alveolitis, unspecified alveolitis, sleep apnoea and respiratory failure. Participants were considered to have cardiovascular conditions if they had reported angina, heart attack/myocardial infarction, heart failure/pulmonary oedema, heart arrhythmia, cardiomyopathy, atrial fibrillation, stroke, ischaemic stroke, transient ischaemic attack or peripheral vascular disease. Presence of myocardial infarction was ascertained using the ICD 10 codes from hospital episodes statistics. Physical activity level quantified as overall average acceleration in milli-gravity was measured with a wrist-worn triaxial accelerometer (Axivity AX3, York, UK). Participants were

instructed to wear the accelerometer on the wrist of the dominant hand for 7 consecutive days. The device data was calibrated and processed to derive the overall acceleration average over a seven-day period by the physical activity expert working group for UK Biobank.

Further details of the study protocol are available online. [www.ukbiobank.ac.uk/resources]

Rubin's rules

The rules developed by Rubin allow combination of parameters of interest (e.g., regression coefficient) from each imputed dataset into an overall estimate while accounting for the extra variability introduced by imputation [1,2]. The following steps described calculation of combined regression coefficient and its variance from multiple imputed datasets.

Combining regression coefficients:

$$\overline{Q} = \frac{1}{m}\sum_{i=1}^{m}\widehat{Q_\iota}$$

where $\overline{Q}$ *= combined regression coefficient, m = total number of imputed datasets,* $\widehat{Q_\iota}$ *= regression coefficient of each imputed dataset*

Combining variance:

$$T = \bar{U} + \left(1 + \frac{1}{m}\right)B$$

where T = combined variance, m = total number of imputed datasets and

$$\bar{U} = \frac{1}{m}\sum_{i=1}^{m}U_i \text{ and } B = \frac{1}{m-1}\sum_{i=1}^{m}(\widehat{Q_\iota} - \bar{Q})^2$$

$$\text{where } \bar{U} = \text{within imputation variance and } B = \text{between imputation variance}$$

Supplemental Figures and Figure Legends

Figure 1. Distribution of hypokinetic segments in AHA 16-segment model for 33 cases with regional wall motion abnormalities

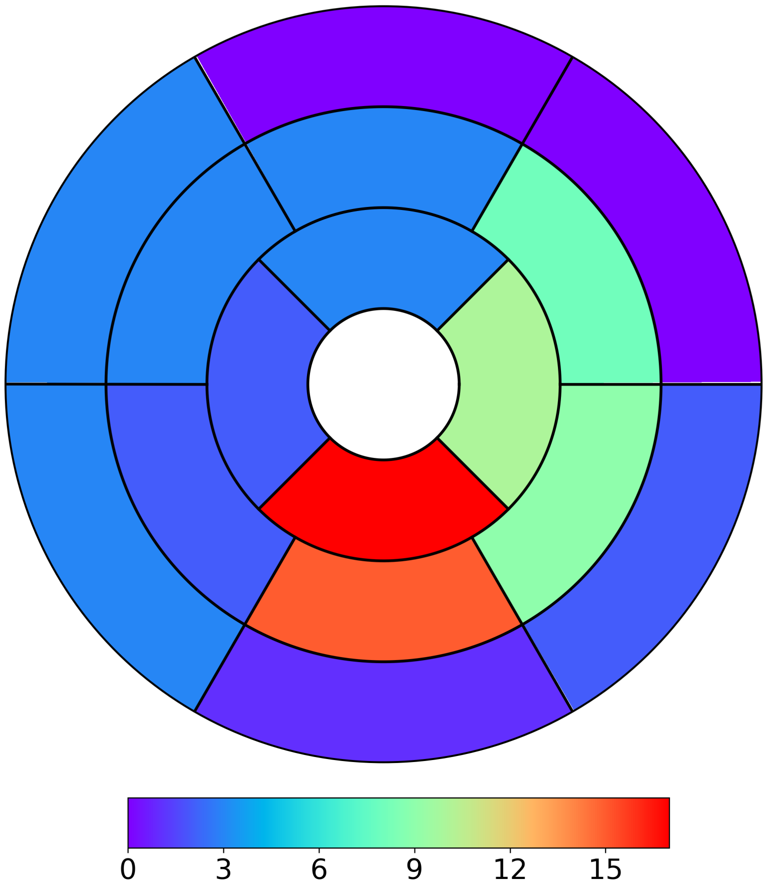

The colour scale at the bottom represents the number of affected cases. Mid to apical inferior and lateral segments were most commonly affected.

Figure 2. Association between restricted-cubic-spline-transformed $PM_{2.5}$ and NO_2 and cardiac parameters

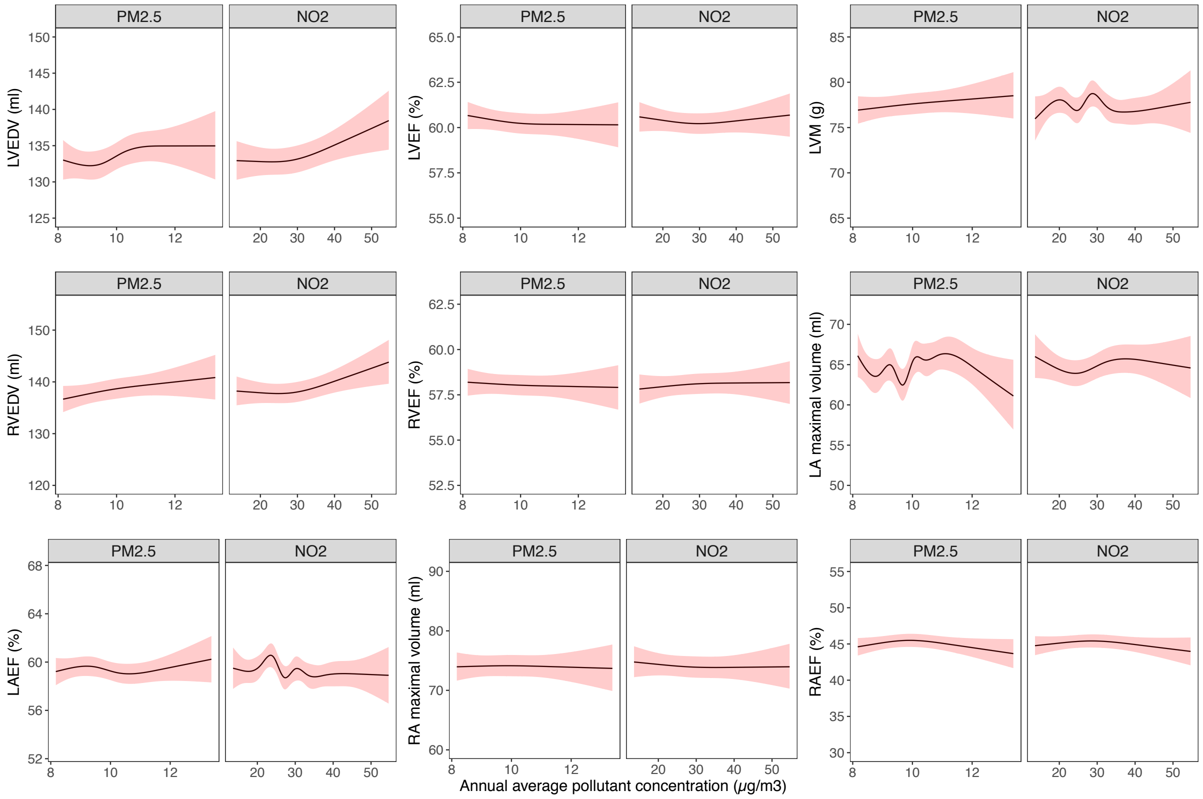

The line and the shaded area represent the predicted mean and 95% confidence interval of cardiac parameters. The test for non-linearity was negative for all except for the associations between $PM_{2.5}$ and LA maximal volume and RAEF. However, the plots did not demonstrate a biologically plausible non-linear relationship.

LV, left ventricle; RV, right ventricle; LA, left atrium; RA, right atrium; EDV, end-diastolic volume; EF, ejection fraction

Supplemental Tables

Table 1. Comparison of participant characteristics between the entire cohort and complete cases

	Entire cohort (N=3,920)	Complete cases (N=1,906)	p-value	Missing, N (%)
Demographics				
Age, years	61.7 (7.4)	61.2 (7.4)	0.004	0 (0)
Male sex	1787 (45.6)	856 (44.9)	0.647	0 (0)
Caucasian ethnicity	3805 (97.1)	1854 (97.3)	0.721	0 (0)
Height, cm	169.5 (9.4)	169.7 (9.2)	0.484	0 (0)
Weight, kg	75.1 (15.1)	74.7 (14.7)	0.323	0 (0)
BMI, kg/m^2	26.6 (4.3)	26.4 (4.3)	0.107	0 (0)
Average household income			0.988	390 (9.95)
< £18,000	480 (13.6)	250 (13.1)		
£18,000 to £30,999	1036 (29.3)	559 (29.3)		
£31,000 to £51,999	1054 (29.9)	579 (30.4)		
£52,000 to £100,000	750 (21.2)	404 (21.2)		
> £100,000	210 (5.9)	114 (6.0)		
Townsend deprivation index	-2.00 (2.65)	-1.97 (2.66)		1 (0.03)
Degree-level or professional education	2495 (63.6)	1263 (66.3)	0.054	0 (0)
Employment status			0.744	5 (0.13)
Skilled job	3097 (79.1)	1525 (80.0)		
Unskilled job	693 (17.7)	319 (16.7)		

Unemployed	121 (3.1)	61 (3.2)		
Retired	4 (0.1)	1 (0.1)		
Clinical characteristics				
Systolic blood pressure, mmHg	137 (18)	136 (18)	0.039	1 (0.03)
Diastolic blood pressure, mmHg	79 (10)	78 (10)	0.019	1 (0.03)
Heart rate, bpm	71 (12)	70 (11)	0.021	0 (0)
Hypertension	1108 (28.3)	518 (27.2)	0.402	0 (0)
Dyslipidaemia	866 (22.1)	400 (21.0)	0.354	0 (0)
Diabetes mellitus	175 (4.5)	89 (4.7)	0.775	0 (0)
Antihypertensive medication	786 (20.1)	368 (19.3)	0.527	0 (0)
Lipid-lowering medication	718 (18.3)	324 (17.0)	0.232	0 (0)
Insulin	25 (0.6)	19 (1.0)	0.185	0 (0)
Smoking status			0.914	9 (0.23)
Never	2398 (61.3)	1168 (61.3)		
Previous	1342 (34.3)	659 (34.6)		
Current	171 (4.4)	79 (4.1)		
Regular alcohol use (≥ 3 times per week)	1757 (44.8)	846 (44.4)	0.769	1 (0.03)
Seven-day average acceleration, milli-gravity	28.19 (9.17)	28.57 (9.32)	0.175	1525 (38.9)
Cardiac phenotypes				
LV EDV, ml	142.3 (33.0)	143.8 (32.2)	0.106	120 (3.06)

LV ESV, ml	58.1 (18.3)	58.8 (17.7)	0.179	120 (3.06)
LV SV, ml	84.2 (19.3)	85.0 (19.1)	0.138	120 (3.06)
LV EF, %	59.5 (6.2)	59.4 (6.1)	0.514	120 (3.06)
LV mass, g	88.4 (24.0)	88.3 (23.4)	0.864	120 (3.06)
LV remodelling patterns			0.395	120 (3.06)
Normal	3504 (92.2)	1758 (92.2)		
Concentric remodelling	140 (3.7)	67 (3.5)		
Eccentric hypertrophy	123 (3.2)	71 (3.7)		
Concentric hypertrophy	33 (0.9)	10 (0.5)		
RV EDV, ml	151.2 (37.1)	152.7 (36.1)	0.148	125 (3.19)
RV ESV, ml	66.7 (22.3)	67.5 (21.7)	0.189	125 (3.19)
RV SV, ml	84.5 (19.4)	85.2 (19.0)	0.207	125 (3.19)
RV EF, %	56.5 (6.5)	56.3 (6.4)	0.37	125 (3.19)
LA maximal volume, ml	66.7 (20.2)	67.4 (20.4)	0.19	349 (8.9)
LA minimal volume, ml	27.6 (12.1)	27.7 (12.0)	0.575	349 (8.9)
LA SV, ml	39.1 (11.2)	39.7 (11.4)	0.079	349 (8.9)
LA EF, %	59.5 (8.3)	59.7 (8.3)	0.538	349 (8.9)
RA maximal volume, ml	78.4 (25.4)	80.0 (25.3)	0.425	209 (5.33)
RA minimal volume, ml	45.2 (18.1)	45.5 (17.7)	0.61	209 (5.33)
RA SV, ml	33.2 (12.5)	33.5 (12.7)	0.378	209 (5.33)
RA EF, %	42.7 (10.3)	42.7 (10.1)	0.925	209 (5.33)
Ambient pollutants				
$PM_{2.5}^{*}$, µg/m^3	9.9 (1.32)	9.9 (1.30)	0.84	10 (0.26)
PM_{10}^{*}, µg/m^3	18.8 (2.11)	18.8 (2.04)	0.783	10 (0.26)

PM_{coarse}*, µg/m^3	6.1 (0.72)	6.0 (0.71)	0.597	10 (0.26)
$PM_{2.5}$ absorbance (elemental carbon)*, per meter	1.13 (0.29)	1.13 (0.29)	0.382	10 (0.26)
NO_2*, µg/m^3	28.2 (11.4)	28.2 (10.9)	0.899	10 (0.26)
NO_x*, µg/m^3	41.5 (17.1)	41.3 (16.7)	0.997	10 (0.26)
24-hour sound level averaged over 1 year*, dB	54.9 (3.6)	54.8 (3.5)	0.349	10 (0.26)
Distance to the nearest major road*, m	356 (555)	359 (553)	0.91	10 (0.26)
Traffic intensity on the nearest major road per day averaged over 1 year*, vehicles/day	15896 (10947)	15853 (10662)	0.462	10 (0.26)
Duration between exposure estimate and imaging visit*, years	5.2 (0.6)	5.1 (0.6)	0.002	0 (0)

Numbers are mean (SD) or number (%), unless otherwise stated. BMI, body mass index; LV, left ventricle; RV, right ventricle; LA, left atrium; RA, right atrium; EDV, end-diastolic volume; ESV, end-systolic volume; SV, stroke volume; EF, ejection fraction; MVR, mass to volume ratio.

*Indicates data presented as median (interquartile range)

Table 2. Associations between annual average particulate matter concentration and cardiac phenotypes

Phenotype	$PM_{2.5}$ (per IQR [1.32 μg/m^3] change)		PM_{10} (per IQR [2.11 μg/m^3] change)		PM_{coarse} (per IQR [0.72 μg/m^3] change)		$PM_{2.5}$ absorbance (per IQR [0.29 m^{-1}] change)	
	Effect size [95% CI]	P-value	Effect size [95% CI]	P-value	Effect size [95% CI]	P-value	Effect size [95% CI]	P-value
LV EDV*	0.83 [0.06 - 1.61]	**0.036**	0.74 [0.01 - 1.47]	**0.048**	0.45 [0.03 - 0.87]	0.034	0.45 [-0.19 - 1.09]	0.17
LV ESV*	1.31 [0.16 - 2.48]	**0.025**	1.06 [-0.02 - 2.16]	**0.055**	0.55 [-0.08 - 1.17]	0.087	0.4 [-0.54 - 1.35]	0.403
LV EF	-0.17 [-0.46 - 0.12]	0.249	-0.12 [-0.39 - 0.15]	0.397	-0.04 [-0.2 - 0.11]	0.582	0 [-0.23 - 0.24]	0.973
LV mass*	0.24 [-0.58 - 1.07]	0.568	0.17 [-0.61 - 0.95]	0.673	0.26 [-0.19 - 0.71]	0.256	-0.01 [-0.68 - 0.67]	0.986
RV EDV*	0.87 [0.1 - 1.65]	**0.026**	0.73 [0 - 1.46]	0.05	0.35 [-0.07 - 0.77]	0.105	0.47 [-0.17 - 1.12]	0.15
RV ESV*	1.2 [0.05 - 2.35]	**0.041**	0.73 [-0.35 - 1.82]	0.186	0.44 [-0.18 - 1.06]	0.166	0.57 [-0.37 - 1.52]	0.237
RV EF	-0.1 [-0.39 - 0.19]	0.501	0.03 [-0.24 - 0.3]	0.846	-0.04 [-0.2 - 0.11]	0.592	-0.04 [-0.28 - 0.19]	0.713
LA maximal volume*	0.55 [-0.8 - 1.92]	0.426	0.32 [-0.93 - 1.59]	0.613	0.35 [-0.37 - 1.07]	0.339	0.84 [-0.26 - 1.95]	0.135
LA EF	-0.08 [-0.49 - 0.33]	0.708	-0.15 [-0.53 - 0.23]	0.431	-0.05 [-0.27 - 0.16]	0.634	-0.1 [-0.42 - 0.23]	0.563
RA maximal volume*	-0.19 [-1.47 - 1.11]	0.777	-0.85 [-2.03 - 0.35]	0.164	-0.5 [-1.18 - 0.19]	0.159	-0.51 [-1.54 - 0.54]	0.34
RA EF	-0.12 [-0.59 - 0.36]	0.633	-0.12 [-0.56 - 0.33]	0.606	-0.02 [-0.28 - 0.23]	0.859	0.2 [-0.19 - 0.6]	0.308
LV geometric patterns								
Concentric remodelling†	1 [0.14 - 6.94]	0.999	1 [0.14 - 6.93]	0.999	1 [0.14 - 6.89]	0.996	1.04 [0.15 - 7.21]	0.968

Eccentric hypertrophy†	1.06 [0.64 - 1.74]	0.831	0.93 [0.58 - 1.48]	0.751	0.83 [0.6 - 1.16]	0.282	0.95 [0.63 - 1.44]	0.816
Concentric hypertrophy†	0.97 [0.43 - 2.19]	0.942	1.08 [0.48 - 2.43]	0.856	1.12 [0.5 - 2.52]	0.788	1 [0.45 - 2.26]	0.992

All estimates were adjusted for the same covariates as the main model except for systolic and diastolic blood pressure, heart rate, hypertension and diabetes.

*log-transformed dependent variables – their effect estimates represent percentage change per IQR increase in exposure variable.

†The effect estimates for these variables represent the odds ratio, where reference is normal LV geometry.

IQR, interquartile range; CI, confidence interval; LV, left ventricle; RV, right ventricle; LA, left atrium; RA, right atrium; EDV, end-diastolic volume; ESV, end-systolic volume; SV, stroke volume; EF, ejection fraction

Table 3. Associations between annual average nitrogen dioxide and nitrogen oxides concentration and cardiac phenotypes

Phenotype	NO_2 (per IQR [11.4 µg/m^3] change)		NO_x (per SD [17.1 µg/m^3] change)	
	Effect size [95% CI]	P-value	Effect size [95% CI]	P-value
LV EDV*	0.88 [0.04 - 1.73]	**0.04**	0.6 [-0.13 - 1.33]	0.109
LV ESV*	0.95 [-0.29 - 2.22]	0.135	1.02 [-0.07 - 2.12]	0.066
LV EF	-0.03 [-0.34 - 0.28]	0.836	-0.15 [-0.43 - 0.12]	0.268
LV mass*	-0.57 [-1.46 - 0.32]	0.206	-0.05 [-0.83 - 0.74]	0.903
RV EDV*	0.86 [0.02 - 1.71]	**0.045**	0.56 [-0.17 - 1.29]	0.133
RV ESV*	0.8 [-0.45 - 2.05]	0.211	0.88 [-0.19 - 1.97]	0.107
RV EF	0.06 [-0.25 - 0.38]	0.685	-0.12 [-0.39 - 0.15]	0.398
LA maximal volume*	0.64 [-0.81 - 2.11]	0.392	0.39 [-0.88 - 1.67]	0.549
LA EF	-0.37 [-0.82 - 0.07]	0.103	-0.22 [-0.6 - 0.17]	0.277
RA maximal volume*	-0.62 [-2 - 0.79]	0.388	-0.42 [-1.62 - 0.79]	0.496
RA EF	-0.17 [-0.69 - 0.34]	0.509	-0.15 [-0.6 - 0.29]	0.499
LV geometric patterns				
Concentric remodelling†	0.91 [0.13 - 6.32]	0.926	0.9 [0.13 - 6.25]	0.916
Eccentric hypertrophy†	0.98 [0.57 - 1.68]	0.952	1 [0.63 - 1.59]	0.994
Concentric hypertrophy†	0.82 [0.37 - 1.85]	0.64	0.89 [0.4 - 2.01]	0.785

All estimates were adjusted for the same covariates as the main model except for systolic and diastolic blood pressure, heart rate, hypertension and diabetes.
*log-transformed dependent variables – their effect estimates represent percentage change per IQR increase in exposure variable.
†The effect estimates for these variables represent the odds ratio, where reference is normal LV geometry.
IQR, interquartile range; CI, confidence interval; LV, left ventricle; RV, right ventricle; LA, left atrium; RA, right atrium; EDV, end-diastolic volume; ESV, end-systolic volume; SV, stroke volume; EF, ejection fraction

Table 4. Associations between annual average 24-hour sound level, distance to nearest major road and annual average traffic intensity on the nearest major road over 24 hour and cardiac phenotypes

Phenotype	Average 24-hour sound level (per IQR [3.6 dB] change)		Distance to the nearest major road (per IQR [555 m] change)		Average traffic intensity (per IQR [10947 vehicles/24h] change)	
	Effect size [95% CI]	P-value	Effect size [95% CI]	P-value	Effect size [95% CI]	P-value
LV EDV*	0.38 [-0.07 - 0.84]	0.098	-0.39 [-0.92 - 0.15]	0.159	0.2 [-0.07 - 0.46]	0.148
LV ESV*	0.73 [0.06 - 1.41]	**0.033**	-0.97 [-1.76 - -0.17]	**0.018**	0.04 [-0.35 - 0.43]	0.831
LV EF	-0.13 [-0.3 - 0.04]	0.125	0.22 [0.02 - 0.42]	**0.034**	0.06 [-0.04 - 0.15]	0.261
LV mass*	0.37 [-0.1 - 0.85]	0.126	-0.61 [-1.19 - -0.03]	**0.039**	0.11 [-0.17 - 0.39]	0.424
RV EDV*	0.08 [-0.37 - 0.53]	0.73	-0.6 [-1.13 - -0.06]	**0.03**	0.19 [-0.07 - 0.45]	0.161
RV ESV*	0.33 [-0.34 - 1]	0.337	-1.01 [-1.8 - -0.22]	**0.013**	0.12 [-0.27 - 0.52]	0.535
RV EF	-0.1 [-0.27 - 0.06]	0.228	0.17 [-0.03 - 0.38]	0.092	0.03 [-0.07 - 0.12]	0.605
LA maximal volume*	0.22 [-0.57 - 1.01]	0.592	-0.21 [-1.15 - 0.73]	0.658	0.07 [-0.39 - 0.52]	0.78
LA EF	-0.07 [-0.31 - 0.18]	0.597	0.12 [-0.17 - 0.4]	0.431	-0.01 [-0.15 - 0.12]	0.842
RA maximal volume*	-0.09 [-0.83 - 0.67]	0.823	-0.31 [-1.21 - 0.6]	0.502	-0.28 [-0.71 - 0.16]	0.216
RA EF	-0.05 [-0.33 - 0.22]	0.7	-0.05 [-0.39 - 0.28]	0.754	0.05 [-0.11 - 0.21]	0.551
LV geometric patterns						
Concentric remodelling†	1 [0.14 - 6.95]	0.997	1.01 [0.15 - 7.01]	0.991	1 [0.14 - 6.95]	0.997
Eccentric hypertrophy†	0.95 [0.7 - 1.28]	0.726	0.92 [0.63 - 1.36]	0.686	0.82 [0.61 - 1.1]	0.184
Concentric hypertrophy†	0.98 [0.44 - 2.22]	0.971	1.07 [0.47 - 2.4]	0.875	1.09 [0.48 - 2.45]	0.837

All estimates were adjusted for the same covariates as the main model except for systolic and diastolic blood pressure, heart rate, hypertension and diabetes.

*log-transformed dependent variables – their effect estimates represent percentage change per IQR increase in exposure variable.

†The effect estimates for these variables represent the odds ratio, where reference is normal LV geometry.

IQR, interquartile range; CI, confidence interval; LV, left ventricle; RV, right ventricle; LA, left atrium; RA, right atrium; EDV, end-diastolic volume; ESV, end-systolic volume; SV, stroke volume; EF, ejection fraction

Supplemental References

1. Rubin, D. B. Multiple imputation for nonresponse in surveys. New York, NY: John Wiley & Sons; 1987.

2. Marshall A, Altman DG, Holder RL, Royston P. Combining estimates of interest in prognostic modelling studies after multiple imputation: current practice and guidelines. *BMC Med Res Methodol*. 2009;9:57.
